# Supplementary material for: Andean agriculture and hand tools: A qualitative approach of exploration of needs, barriers, and opportunities for innovation
Source: PLoS One. 2026 May 15;21(5):e0335295. doi: 10.1371/journal.pone.0335295 (PMC13178989; doi:10.1371/journal.pone.0335295)
Supplement: S1 File — (DOC) [file pone.0335295.s001.doc]

**Supplemental file 5**.

Interview Guide on Needs, Perceptions, Barriers, and Facilitators (First guide – Adjusted)

Good morning, Mr./Ms. ___________________. Could you please tell me your full name and age?

Thank you, Mr./Ms. ____________. I’m going to ask you some questions about potato harvesting, particularly about the activity of digging up the potatoes and the tools you use for that. Please remember that there are no right or wrong answers — any information you provide is very valuable to us.

**Experiences, perceptions, and needs**

1. What are your main economic activities? (introduction)
2. How long does it take you to get from your home to the harvesting site? (What is the road like? Is access easy or difficult? Do you use animals for transport or to carry loads?) What is the terrain like where you harvest?
3. How do you transport your harvesting tools? (Who carries them? How are they carried?)
4. What tools do you currently use for digging up potatoes during harvest? (What are they called? Please describe them.)
5. Is there any particular tool you prefer to use over the others? Why do you prefer these tools? What do you like most about them? (weight, shape, size, material)
6. Do you think the weather (for example, sun, rain, or humidity) affects the condition or durability of these tools? How so?
7. How do you use your tool? (Simulate holding and using it.) Do you feel comfortable using it that way? (Explore each tool mentioned by the participant.)
8. Which parts of your body feel most tired or sore during the digging activity? (Back, hands, arms.) (Explore each tool mentioned by the participant.) Do you think that the pain or tiredness after the activity is related to the tool you use?

**Barriers and facilitators**

1. Have you always used that tool, or did you use another one before? What made you change? Why do you prefer this tool? Since when have you used it? How did you learn to use it? (Did someone teach you, or did you learn by observing others?)
2. If you had access to a new harvesting tool, what difficulties do you think it could help solve? What would you like it to be like? (Show cards or images of tool parts: handle, head, size, weight.) (Provide a sheet of paper for the participant to draw how they would like the tool to be.)
3. While the participant draws, probe further:
4. How heavy or light would you like it to be? Why?
5. What kind of material do you think would work best in this area? (wood, metal, other)
6. What color would you choose? Is that important to you?
7. How should it feel when you use it? (For example: comfortable, fast, safe…)
8. What should a good tool avoid? (What would you not like it to have?)
9. If they say no, follow up: Why not? (Explore motivations: cost, fear, need for training.)

**Additional closing questions**

1. What does agriculture mean to you?
2. Have you always wanted to work in this activity?

**Annex 6**. Interview Guide on Perceived Satisfaction in the Use of the Traditional Tool for Tuber Harvesting (Second guide)

Good morning, Mr./Ms. __________________. I hope you had a productive day harvesting. I’d like to take a few minutes to ask you some questions about your experience today.

1. Was there any moment during the harvest when you felt that the tool didn’t work as expected? Perhaps a problem that required repair or adjustment before using it again?
2. During your work, were there moments when the tool felt uncomfortable or difficult to handle?
3. How did you find the weight, size, or shape of the tool while working?
4. Today, after using the tool, how would you describe its condition? Do you feel it’s still in good shape, or have you noticed any signs of wear?
5. What kind of maintenance do you think it will need? What’s your opinion about how easy or difficult it will be to carry out that maintenance?
6. Now that you’ve finished your workday, how would you describe your overall satisfaction with the tool you used today?

Thank you very much for your time and for sharing your experience with us.
