# Supplementary material for: Andean agriculture and hand tools: A qualitative approach of exploration of needs, barriers, and opportunities for innovation
Source: PLoS One. 2026 May 15;21(5):e0335295. doi: 10.1371/journal.pone.0335295 (PMC13178989; doi:10.1371/journal.pone.0335295)
Supplement: S2 File — (DOC) [file pone.0335295.s002.doc]

| N° | item | Guide questions/ description | statement |
| --- | --- | --- | --- |
|  | **Domain 1. Research team and reflexivity** | |  |
|  | *Personal characteristics* |  |  |
| 1 | Interviewer/facilitator | Which author/s conducted the interview or focus group? | Enma Maco |
| 2 | Credentials | What were the researcher’s credentials? E.g. PhD, MD | B.Sc. in Psychology |
| 3 | Occupation | What was their occupation at the time of the study? | She was working as a licensed psychologist |
| 4 | Gender | Was the researcher male or female? | Female |
| 5 | Experience and training |  |  |
|  | *Relationship with participants* |  |  |
| 6 | Relationship established | Was a relationship established prior to study commencement? | Yes, a meeting was held with a farmers’ representative to present the study purpose |
| 7 | Participants knowledge of the interviewer | What did the participants know about the researcher? e.g. personal goals, reasons for doing the research | Participants were told about study goals. |
| 8 | Interviewer characteristics | What characteristics were reported about the interviewer/facilitator? e.g. Bias, assumptions, reasons and interests in the research topic | The interviewer had professional experience in the field and demonstrated awareness of her potential influence on the research |
|  | **Domain 2: study design** |  |  |
|  | *Theoretical framework* |  |  |
| 9 | Methodological orientation and theory | What methodological orientation was stated to underpin the study? e.g. grounded theory, discourse analysis, ethnography, phenomenology, content analysis | phenomenology |
|  | *Participant selection* |  |  |
| 10 | Sampling | How were participants selected? e.g. purposive, convenience, consecutive, snowball | Purposive sampling |
| 11 | Method of approach | How were participants approached? e.g. face-to-face, telephone, mail, email | Face-to-face |
| 12 | Sample size | How many participants were in the study? | 13 participants |
| 13 | Non-participant | How many people refused to participate or dropped out? Reasons? | One participant missed the second interview because we were unable to locate them during the harvest in the field and neither their home. |
|  | Setting |  |  |
| 14 | Setting of data collection | Where was the data collected? e.g. home, clinic, workplace | Workplace and home |
| 15 | Presence of non-participants | Was anyone else present besides the participants and researchers? | The interpreter |
| 16 | Description of sample | What are the important characteristics of the sample? e.g. demographic data, date | Farmers of 30 years or older and having at least six months of continuous engagement in agricultural work. Interviews were carried out from may 13^th^ to may 24^th^ |
|  | *Data collection* |  |  |
| 17 | Interview guide | Were questions, prompts, guides provided by the authors? Was it pilot tested? | Yes, it was piloted with one participant. |
| 18 | Repeat interviews | Were repeat interviews carried out? If yes, how many? | No repeated interviews were carried out. |
| 19 | Audio/visual recording | Did the research use audio or visual recording to collect the data? | Audio recording |
| 20 | Field notes | Were field notes made during and/or after the interview or focus group? | Yes, the interviewer took field notes. |
| 21 | Duration | What was the duration of the interviews or focus group? | Yes, interviews lasted 30–40 minutes; the second, 15–20 minutes |
| 22 | Data saturation | Was data saturation discussed? | Yes, data saturation was reached after the 11th interview |
| 23 | Transcripts returned | Were transcripts returned to participants for comment and/or correction? | No |
|  | **Domain 3: analysis and findings** |  |  |
| 24 | Number of data coders | How many data coders coded the data? | Two coders (LCA, EMC) |
| 25 | Description of the coding tree | Did authors provide a description of the coding tree? | yes |
| 26 | Derivation of themes | Were themes identified in advance or derived from the data? | We based identification themes on Jain´s work (task, human, product and qualitative considerations) Later, and additional theme emerged from data (environmental) |
| 27 | Software | What software, if applicable, was used to manage the data? | Yes, we use Atlas.Ti 25 |
| 28 | Participants checking | Did participants provide feedback on the findings? | no |
|  | *Reporting* |  |  |
| 29 | Quotations presented | Were participant quotations presented to illustrate the themes / findings? Was each quotation identified? e.g. participant number | Yes, quotations are stated in main text and additional quotations in supplementary material |
| 30 | Data and findings consistent | Was there consistency between the data presented and the findings? | yes |
| 31 | Clarity of major themes | Were major themes clearly presented in the findings? | Yes, the major themes are clearly presented, supported with participant quotations, and further explained. |
| 32 | Clarity of minor themes | Is there a description of diverse cases or discussion of minor themes? | no |
