# Supplementary material for: Andean agriculture and hand tools: A qualitative approach of exploration of needs, barriers, and opportunities for innovation
Source: PLoS One. 2026 May 15;21(5):e0335295. doi: 10.1371/journal.pone.0335295 (PMC13178989; doi:10.1371/journal.pone.0335295)
Supplement: S3 File — (DOCX) [file pone.0335295.s003.docx]

Inclusivity in global research

PLOS’ policy on inclusivity in global research aims to improve transparency in the reporting of research performed outside of researchers’ own country or community and ensures that PLOS publications reporting global research adhere to high standards for research ethics and authorship. Authors of relevant research articles may be asked to complete the questionnaire below, which outlines ethical, cultural, and scientific considerations specific to inclusivity in global research. This questionnaire may be requested when researchers have travelled to a different country to conduct research, if research uses samples collected in another country, research with Indigenous populations or their lands, or if research is on cultural artefacts. Researchers travelling to another country solely to use laboratory equipment will not normally be required to complete the questionnaire. However, the questionnaire can be requested at the journal’s discretion for any submission – if you have been requested to complete this questionnaire by the PLOS journal you submitted to, please do so.

Please complete the questionnaire below and include this as a Supporting Information file with your manuscript. Note that if your paper is accepted for publication, this checklist will be published with your article in the supporting information files. Please ensure that you reference the checklist in the main body of your manuscript. We suggest adding a subsection ‘Inclusivity in global research’ to your Methods section and adding the following sentence: “Additional information regarding the ethical, cultural, and scientific considerations specific to inclusivity in global research is included in the Supporting Information (SX Checklist)”

The questions have been designed to be applicable to a wide range of study types, and there are subsections for both human subjects research and non-human subjects research. If any of the questions are not relevant to your research please mark them as “N/A” as appropriate.

**Ethical considerations, permits and authorship**

*This section is applicable to all research types.*

Provide details as to who granted permissions and/or consent for the study to take place in the Methods section of your manuscript. This should include the names of **all** ethics boards, governmental organizations, community leaders or other bodies that provided approval for the study. If individuals provided approval refer to these people by their role or title but do not list their name(s).

Reported on page number: Yes. The study was approved by IRB of Universidad Nacional Mayor de San Marcos, Perú (study code: 0072-2025). Permissions were obtained from DIRESA–Apurímac (Regional Health Directorate of Apurímac) via Letter No. 004-2025-CRPDI-DEGDRH-DIRESA-AP (Registration No. 1687), and from the local community through the community president. The community president is the recognized representative of the community; permission was granted after a meeting where the study was explained and was documented in writing by the president’s signature on the university letter of introduction supporting the Principal Investigator. Page n°5

If there were any deviations from the study protocol after approval was obtained please provide details of these changes in the Methods section of your manuscript.
Did this study involve local collaborators that are residents of the country where the research was conducted or members of the community studied? If you do not have any authors from said communities, please provide an explanation for this below.

Yes. Local collaboratorion was included. A resident of the study area, with established relationships and deep knowledge of local farmers, participated as a community liaison during the study’s outreach engagement phase. She helped with community presentations and supported the invitation and recruitment of farmers. This collaboration enabled a culturally appropriate approach and effective communication.

Reported on page number: None deviations from the established protocol. Page n°5.

Everyone listed as an author should meet PLOS’ criteria for authorship and all individuals who meet these criteria should be included in the author byline, rather than the acknowledgements. For further information please see the journal’s Authorship Policy.

**Human subjects research (e.g. health research, medical research, cross-cultural psychology)**

Did you obtain written informed consent from a representative of the local community or region before the research took place? How did you establish who speaks for the community? Details of written informed consent obtained from study participants should be reported separately in the Methods section of your manuscript.

Yes. Prior to data collection, we obtained written authorization at the regional level from DIRESA–Apurímac (Regional Health Directorate of Apurímac), which approved the project through Letter No. 004-2025-CRPDI-DEGDRH-DIRESA-AP (Registration No. 1687). At the community level, the study was presented in a meeting with the community president, and the community’s permission to conduct the research was documented in writing: the community president signed the official university letter of introduction endorsing and supporting the Principal Investigator, thereby providing written community authorization before the research took place. We established who speaks for the community by consulting the community’s governance structure. Written informed consent from individual study participants is reported separately in the Methods section.

How did members of the local community provide input on the aims of the research investigation, its methodology, and its anticipated outcome(s)?

When engaging with the local community, how did you ensure that the informed consent documents and other materials could be understood by local stakeholders?

To ensure that informed consent materials and other study documents were understandable to local stakeholders, the guides and consent-related materials were developed in Spanish, using clear, non-technical language, and were checked during fieldwork to confirm clarity and appropriateness in the local context. Because many community members speak both Spanish and Quechua, participants were also offered the option to communicate in Quechua. When a participant preferred Quechua, a bilingual interpreter supported the process by translating the study information and facilitating translation of questions and responses to ensure accurate understanding and communication

The protocol was finalized before fieldwork, so community members did not contribute to changing the study aims or methodology. Their input was limited to implementation: during presentations and recruitment, questions/feedback helped refine outreach messaging and logistics (eg., culturally appropriate invitations and communication)supported by a local resident collaborator.

Will the findings of the research be made available in an understandable format to stakeholders in the community where the study was conducted (e.g. via a presentation, summary report, copies of publications, etc.)? Please provide details of how this will be achieved.

Yes. After the analysis, we will hold an in-person presentation with the agricultural community to share the key findings in clear, non-technical language, with time for questions. If needed, Spanish–Quechua bilingual support will be provided.

**Non-human subjects research using specimens/ animals collected as part of the study, or those housed in archival collections. Examples include archaeology, paleontology, botany and zoology.**

Did the permission you obtained from a local authority to perform the study include an agreement on access to outputs and benefit sharing? This may include procedures to enable fair distribution of the benefits and resources arising from the research performed. Please include any details of Prior Informed Consent and Benefit Sharing Agreements obtained. These may be required by field-specific regulations, for example the Convention on Biological Diversity (CBD) and the associated Nagoya Protocol.

N/A

If the material used in your study was imported, please A) provide the year it was imported and B) indicate whether permits were obtained to import/export the materials used, C) provide details of any permits obtained. If this information is not available, please indicate this.

N/A

If you used archival specimens, please state how the material used in your study was acquired by the institute it is held in and provide details of any permits obtained for the original excavations/ sample collection. If this information is not available, please indicate this.

N/A

How was the potential cultural significance of the materials collected in your study to local communities considered in your research design? Were Indigenous peoples and/or local researchers and institutions involved with archaeological excavations / collection of specimens? If so, please provide a description of their involvement.

N/A

If your manuscript includes photographs of human remains please indicate whether authors obtained permission from descendants or affiliated cultural communities to do so.

N/A
