# Supplementary material for: Andean agriculture and hand tools: A qualitative approach of exploration of needs, barriers, and opportunities for innovation
Source: PLoS One. 2026 May 15;21(5):e0335295. doi: 10.1371/journal.pone.0335295 (PMC13178989; doi:10.1371/journal.pone.0335295)
Supplement: S4 File — (DOCX) [file pone.0335295.s004.docx]

**Supplemental file 4**

**Table 1**. Themes, codes of representative quotes of first guide

| **Themes** | **Codes** | **Representative quotes** |
| --- | --- | --- |
| Task | Work terrain conditions | "Our land it´s uphill, quite steep" (p1, woman, 53 years old). "Our land is a bit difficult...the access is through slopes" (p9, man, 62 years old). "That part of land doesn´t have water. Only with rain the potatoes, beans, beans, wheat, and oca can grow there) (p10, woman, 43 years old). "Where you plant potatoes is communal land. You can farm it this year, the next, three, four, five years, but then the land rests for about five years before it´s rotate to another farmer” (p2, man) |
|  | Travel conditions and means of transport | “Yes, it’s quite far […] you can only go on foot, just people, because there are fences […] but there is another path for the animals lower down” (p9, man) “We carry it on the horse, sometimes on our shoulders…when there is no horse, we carry it on our shoulders…normally we walk ourselves with the tool on our shoulder” (p4, man). |
|  | Time and breaks during transportation | “And hour and a half on foot sometimes…but on horseback it’s a little less, maybe 50 or 60 minutes. At least one hour (by the short route)” (p6, man) " I just sat down for a little while halfway, then kept going straight uphill from there" (p5, woman 50 years old) "it´s about an hour and a half on foot sometimes...but on horseback it´s a bit less" (p6, man, 47 years old) |
|  | Fit for the user | “Because it’s light for my hand, that’s why I use it” (p1, woman, 53 years old) “We mainly use the medium pickaxe, since it doesn’t weigh much. Men use a bigger pickaxe, because of the strength they have” (p11, woman, 33 years old). " Well, each one knows their own body, right? you figure it out...whether to take a small pickaxe or a bigger one " (p10, woman 43 years old) |
| Human | Companions and family dynamics | “I take my children to dig when potato plants begin to die back. I walk, carrying food…bringing them, and when they fall behind, the say ‘Mom, pick me up, pick me up!” (p8, woman,41 years old). "For example, now the kids, like I said before, go to the harvest. Our children go and help, even the little ones are there" (p9, man 62 years old). "That´s how I team up with my brother-in-law: one day for him, one day for me, and we dig like that, all together" (p1, woman, 53 years old) "I go with my partner, and sometimes my brothers help me with that, also my nephews, my uncles, the neighbors." (p11, woman, 33 years old) |
|  | Intergenerational learning | "I learned that by watching my mom and my dad. Just by looking...my mom used to tell me: this is how you work the field" (p1, woman, 53 years old) "Since I was 12 years old I helped my mom [...] watching her and other people digging the land" (p3, man,78 years old). "Wherever it was, my mom would take me with my allachu to help. Since I was about 11 or 12 years old, I already knew how  to earn my own living" (p5, woman, 50 years old). “we´ve been learning since we were 12…there, where our parents farm. You go with your mom to help her…” (p10, woman, 43 years old) |
|  | Transition in tool use | “I spent some time on the Coast. When I came back, there were almost no allachu left. I do not know what happened” (p6, man) “Now it’s modernized to that pickaxe. And that allachu they talk about is disappearing—only a few people probably still have one.” (p11, woman) "When I was around 7 or 10 years old, I still didn´t have the strength, so I had to switch. My dad gave this lighter pickaxe" (p4, man, 55 years old) "Little by little, shovels started to appear for everyone, and now there are no more allachu" (p9, man, 62 years old) "Before, they called it allachu. My dad used it...but now I just use the pickaxe. Around here, we mostly use the pickaxe..." (p12, man, 47 years old) |
|  | Discomfort and bodily pain | "Basically, when you work, your waist or your arms get tired, especially when the soil is very hard...mostly the waist. I think also because you´re bent over the whole time, picking up potatoes" (p13, man, 44 years old" "It´s always, always my lower back that gets tired, mostly in the back here... because you´re always bent over and putting in strength" (p12, man, 47 years old). "When you work constantly, or all day on something heavy, the next day your whole body aches" (p11, woman, 33 years old). “And when I hold the pickaxe, my hand hurts; it hurts when I press like this” (points to the fingers of her right hand), “this is what does all the work.” (p5, woman) |
|  | Processes of bodily adaptation | “Nowadays, I use the pickaxe a lot, and the next day my hands are numb… my lower back…my arms.” (p10, woman) " No, nothing...our legs are already used to it" (p3, man 78 years old) "I come home with back pain...but when you´re used to it every day, it feels normal" (p10, woman, 43 years old)  "The first day it tires you out, but the next day you do it again, and it´s less tiring" ( p11, woman 33 years old) |
|  | Social and technical adoption (barrier and enabler) | “If you use another tool, it’s… uncomfortable, sometimes it feels […] a bit wider, a bit tighter… so sometimes you don’t like it; you prefer to work with your own tool, you, see?” (p6, man, 47 years old). "Here, women sometimes get used to using a medium pickaxe" (p10, woman, 43 years old). "The allachu they mention, in the old days it was used. Now, it´s been replaced by the pickaxe" (p11, woman, 33 years old) |
| Product | Types and functions of the tool | "The allachu is just for digging land and take potatoes, with a single tip" (p1, woman, 53 years old). "The allachu is not as comfortable as the pickaxe; it doesn´t have two points" (p2, woman 49 years old). "The pickaxe is for harvests, we use it to prepare the soil.... the allachu is for open the land, for potatoes" (p3, man, 78 years old) "I mostly work with the small pickaxe, because it´s better for pulling out weeds. The pickaxe has more strength for that, while the allachu doesn´t" (p8, woman, 41 years old) "For digging potatoes we already use the pickaxe. Some people carry small pickaxe...and if not, then a big pickaxe" (p12, man, 44 years old) |
|  | Access to tools | "Right now, I don’t have it, ma’am, my pickaxe [...] My cousin told me: ‘In Abancay, she says she bought her light pickaxe. Yeah, she bought it in Abancay, she says.’" (p5, woman 50 years old). "We also sell them... if someone asks, we just give them one" (p6, man, 47 years old) "I want to buy one. A man comes to the municipality to work, he makes small pickaxes, and I told him: sell me one" (p1, woman, 53 years old) |
|  | Technical characteristics | " We use one pickaxe that´s a bit thinner than the regular pickaxes, for harvest time" , "The *chachacoma* or *tasta* wood are the ones that resist more and last longer" (p6, man, 47 years old)  "The head and the handle must be well adjusted" (p7, woman, 49 years old"  "Normally the pickaxe shouldn't look like this... it should be straight. This one is kind of oval because it's worn out" (p9, man, 62 years old) "if you make a hole, it's because it has more of a point and goes in deeper, so you do it faster"( p11, woman, 33 years old) |
|  | Use, maintenance and repair | "I left it like that, the handle rotted and I had it replaced. When the handle rots, if you strike hard, it breaks" (p1, woman) "For example, the tips wear out and we have to take them to the blacksmith so he can sharpen both sides" (p4, man, 55 years old) "Sometimes when you bury it or leave it somewhere, it gets wet and grows mold...so you just have to replace it" (p6, man) "When it's like that (worn out), we have to take it to the blacksmith. He has to hammer it back and fix its shape" (p9, man, 62 years old) |
|  | Physical characteristics of the tool (Barrier and enabler) | "Whether it´s an allachu or a pickaxe, it should be light enough for your grip, not too heavy" (p5, woman,50 years old) "There are others hand tools that are rough, that make your hand hurt and bother you" , "This pickaxe tip goes it with a single point...sometimes it hits the potato and breaks it. I don't like the width at the back of the tip; during harvest, sometimes it breaks the potatoes" (p6, man 47 years old) "It has to be comfortable, because if it isn’t, you can’t work. Whether it’s small or big, if the handle isn’t well sanded, it makes your hand hurt" (p7, woman, 49 years old). "If you’re going to work with a thick pickaxe, it gets heavier for your hand, right? and you get tired faster." (p10, woman, 43 years old). “the discomfort happens when the small pickaxe isn’t well sanded: the handle makes your hand hurt or can scrape you (p13, man) |
|  | Availability and access (Barrier and enabler) | "A man comes to the municipality to work, he makes pickaxes" (p1, woman 53 years old) “We would have to go to the smithy; that’s where we get the pickaxes, and we would have them made so we can work” (p4, man, 55 years old) "mmm, I had a pickaxe, but I haven´t found it" (p5, woman, 50 years old) "I mean, I don't see the allachu in hardware stores...most people use this type of pickaxe. Nowadays I only see medium ones or small pickaxes in the store", "We use just pickaxe...because here we easily find it, we mostly use pickaxe" (p12, man, 47 years old) |
|  | Ideal weight and size of the tool | "This little pickaxe is very light, very special... if the handle is shorter, you bend more; if it´s longer, you bend a bit less" (p9, man 62 years old) "of course, I had pickaxes that were heavy, made for men, and they were just too much...but when it´s light, yes, it helps you work faster" (p5, woman, 50 years old) "It has to be comfortable... it also has to fit your size"(p7, woman, 49 years old) "I feel comfortable with the medium one...yes, lighter. There are also medium- sized ones that don't weigh much" (p10, woman, 43 years old) |
|  | Shape and design of the head and tip | "The iron tip should be longer...that way it doesn´t damage the potato. Sometimes with the wrong tip you crush it and break it, but with this one, no", " more pointed and longer...that´s why men prefer bigger pickaxes, while women use smaller ones"(p2, woman, 49 years old) "..I wish this could be light, with two head tips but lighter" (p5, woman, 50 years old) "The allachu is also wide. There's no pickaxe that hooks with the point; it's double the work...The tip should be sharp, like this one, ending in a point" (p6, man, 47 years old) "The pickaxe has a double function: the tip and the flat blade" (p9, man, 62 years old) |
|  | Handle characteristics | "The handle must be smooth in this part and have a firm grip, otherwise it gives you blisters" (p7, woman, 49 years old) "Here the handle is already bent; I have to replace it " (p9, man, 62 years old) "Splinter can get into your hand, so it has to have a good surface, well sanded" , "Regarding the handle, if you used iron, it would also weigh more. That’s why I use a Tasta wood handle, which weighs less” (p13, man, 44 years old) |
|  | Preferred material and expected desirability | “With Tasta wood […] this handle lasts […] eucalyptus breaks when you dig… eucalyptus doesn’t last.” (p1, woman) "Since it´s *Tasta* wood, it doesn´t break; it´s a good plant, good wood" (p3, man 78 years old). "Here we use tasta or molle, it's about the same. Both last" (p5, woman 50 years old) " Chachacoma or tasta last longer than eucalyptus, they resist more" (p6, man, 47 years old) Regarding the head material: “Soft iron breaks more quickly when you dig into stone, but regular iron doesn’t break, it just wears down” (p11, woman, 33 years old) |
| Qualitative | Value attributed to tools | "When more time passes, the soil hardens, so there are times when the ground is hard and the pickaxe won't go in, so you use the blade to help yourself" (p9, man 62 years old) "When you want to work faster, you also use a lot of force. That´s when you use the small pickaxe, with the small pickaxe or small allachu" (p10, woman, 43 years old) "When you don't have enough tools, you take the allachu, and then you give them to the people" (p13, man, 44 years old) |
|  | Improvement proposal | "I wish that new tool wouldn't break the potatoes... and maybe also, it could be the machines, so the machine does the digging work" (p13, man, 44 years old) "Lighter, yes that could work, but there's no strength, they don't have weight; the tool always has to have weight" (p9, man 62 years old) "Now it's coming out with three points, one like this (points to the drawing her husband made) and with that you can pull everything, even grass" (p7, woman, 49 years old) “Yes, one meter, a meter and a little longer for the handle” (p4, man, 55 years old).  “Almost pointed, like this one, almost pointed, so that it ends in a point,” (he points to his drawing). And about the handle he said, “…then it would be a bit more curved, but not too much.” (p6, man, 47 years old) “That’s also good if this part has three points” (points to her husband’s drawing- p6), “now they’re coming out with three points, and with that you can pull everything, even the grass” (p7, woman). |
|  | Preferences according to the user | "I mostly use little picks... it feels more comfortable" (p6, man 47 years old) "Yes, since it´s light, I dig quickly, and then with two points, I dig even faster" (p1, woman, 53 years old) "For women, something light, you know. I liked the little pick because it´s light" (p5, woman 50 years old). "There are other pickaxe that are rough on the handle, which make your hand hurt and are uncomfortable, so you don't prefer those" (p6, man 47 years old) |
| Environmental | Effects of climate on the tool | “I left it outside, and it rotted, so I had to replace its handle. It breaks when the handle rots—if you strike it hard, it just snaps” (p1, woman) “The wood swells in the rainy season if its gests wet…in the dry season, no, it´s normal” (p2, woman) “When the allachu or pickaxe is left outside in the rain, it gets wet, turns to mud, mud sticks to it, and you have to clean it […] I always leave it in the sun […] it holds up very well” (p3, man) |
