# Supplementary material for: Andean agriculture and hand tools: A qualitative approach of exploration of needs, barriers, and opportunities for innovation
Source: PLoS One. 2026 May 15;21(5):e0335295. doi: 10.1371/journal.pone.0335295 (PMC13178989; doi:10.1371/journal.pone.0335295)
Supplement: S5 File — (DOC) [file pone.0335295.s005.doc]

**Supplemental file 5**

Table 2 Anthropometric characteristics of participants

| **Anthropometric parameters** |  | **General** | | **Female** | | | | | **Male** | | | |  |
| --- | --- | --- | --- | --- | --- | --- | --- | --- | --- | --- | --- | --- | --- |
|  |  | Mean | SD | 5th | 50th | 95th | Mean | SD | 5th | 50th | 95th | Mean | SD |
| Maximum hand length (mm) |  | 173.9 | 15.9 | 152 | 172 | 206 | 165.2 | 13.7 | 152 | 162.5 | 187 | 182.7 | 13.7 |
| Palm length (mm) |  | 100.2 | 10.5 | 87 | 97.5 | 115 | 94.2 | 9.8 | 87 | 90.5 | 113 | 106.2 | 7.8 |
| Hand breadth (mm) |  | 80.3 | 7.0 | 68 | 82 | 93 | 76.8 | 6.8 | 68 | 76 | 85 | 83.7 | 5.9 |
| Maximum hand breadth (mm) |  | 91.1 | 12.3 | 70 | 91.5 | 113 | 82.2 | 8.7 | 70 | 83 | 94 | 100.0 | 8.1 |
| Maximum grip diameter (mm) |  | 41.5 | 3.2 | 37 | 41.5 | 48 | 39.7 | 2.3 | 37 | 39.5 | 42 | 43.3 | 3.1 |
| Phalanx length – 1^st^ finger (mm) |  | 53.9 | 5.8 | 48 | 52 | 64 | 50.5 | 3.4 | 48 | 49.5 | 57 | 57.3 | 5.9 |
| Phalanx length – 2^nd^ finger (mm) |  | 62.4 | 5.5 | 56 | 61.5 | 74 | 59.2 | 2.5 | 56 | 58.5 | 63 | 65.7 | 5.8 |
| Phalanx length – 3^rd^ finger (mm) |  | 69.8 | 5.6 | 63 | 69 | 81 | 66.7 | 4.0 | 63 | 66 | 73 | 73.0 | 5.4 |
| Phalanx length – 4^th^ finger (mm) |  | 65.1 | 4.4 | 59 | 64.5 | 73 | 62.2 | 2.9 | 59 | 62 | 67 | 68.2 | 3.5 |
| Phalanx length – 5^th^ finger (mm) |  | 50.2 | 5.0 | 44 | 49.5 | 59 | 46.7 | 3.1 | 44 | 46 | 52 | 53.7 | 4.0 |
| Hand circumference (mm) |  | 190.9 | 16.9 | 168 | 189 | 220 | 177.7 | 9.1 | 168 | 177.5 | 189 | 204.2 | 11.2 |
| Maximum hand circumference (mm) |  | 229.2 | 24.7 | 192 | 227.5 | 277 | 210.5 | 14.4 | 192 | 210 | 228 | 247.8 | 17.2 |
| Forearm length (mm) |  | 246.9 | 19.4 | 231 | 239 | 282 | 233.7 | 2.9 | 231 | 233 | 239 | 260.2 | 20.0 |
| Arm length (mm) |  | 301.1 | 29.4 | 270 | 290 | 352 | 282.8 | 15.9 | 270 | 276.5 | 310 | 319.3 | 29.0 |
| Trunk length (mm) |  | 461.5 | 47.6 | 398 | 444 | 554 | 425.8 | 16.8 | 398 | 428 | 442 | 497.2 | 40.5 |

n=12

SD: standard deviation
