# Supplementary material for: Andean agriculture and hand tools: A qualitative approach of exploration of needs, barriers, and opportunities for innovation
Source: PLoS One. 2026 May 15;21(5):e0335295. doi: 10.1371/journal.pone.0335295 (PMC13178989; doi:10.1371/journal.pone.0335295)
Supplement: S6 File — (DOC) [file pone.0335295.s006.doc]

**Supplemental file 6.** Tools and proposals of improvements identified by farmers

**
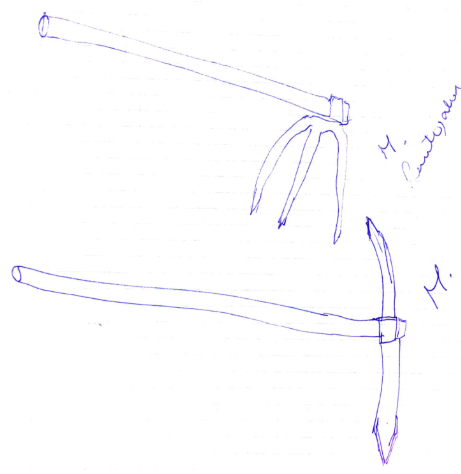
**

Participant 6


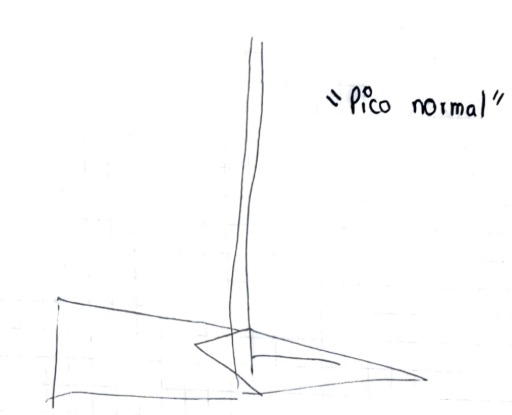


Participant 11


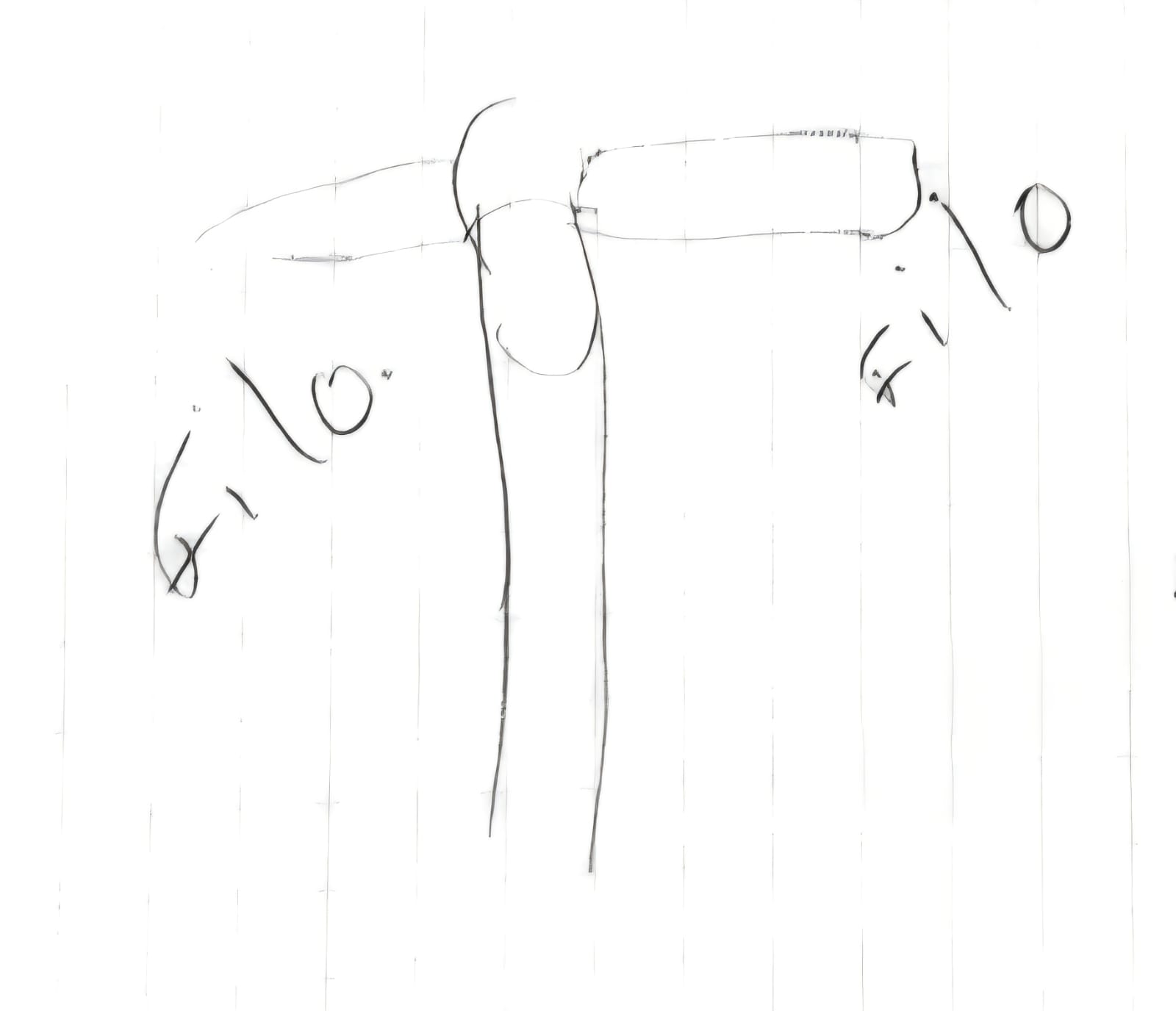


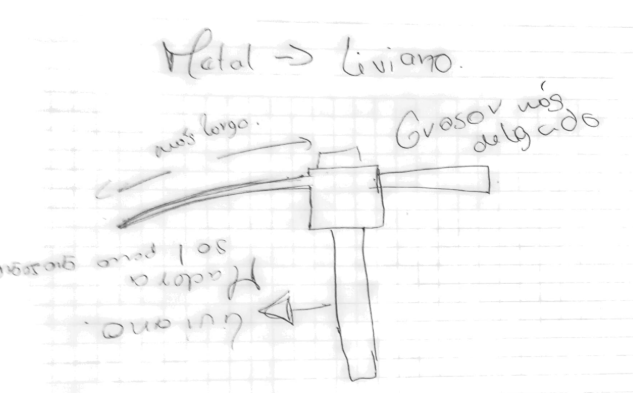
Participant 8

Participant 2


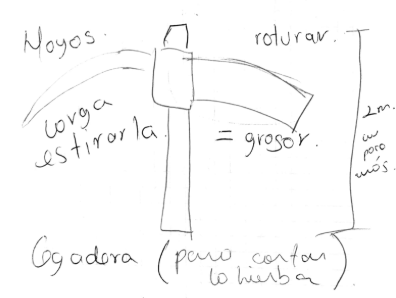


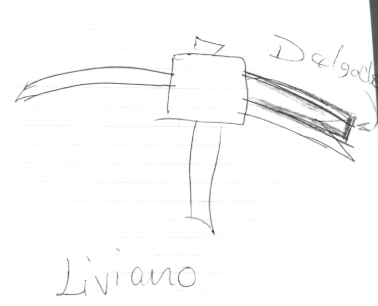
Participant 4

Participant 5.


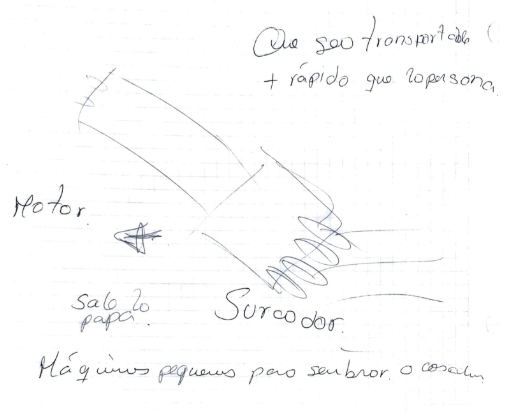
 Participant 13.
