# Supplementary material for: Andean agriculture and hand tools: A qualitative approach of exploration of needs, barriers, and opportunities for innovation
Source: PLoS One. 2026 May 15;21(5):e0335295. doi: 10.1371/journal.pone.0335295 (PMC13178989; doi:10.1371/journal.pone.0335295)
Supplement: S7 File — (DOCX) [file pone.0335295.s007.docx]

**Supplemental file 7**

**Table 3**. Domains, codes and representative quotes of second guide

| **Themes** | **Codes** | **Representative quotes** |
| --- | --- | --- |
| Tools conditions for efficient use | Appropriate weight | "The pickaxe felt easy to handle, it didn’t weigh much… nice and light, normal, comfortable" (p1, woman, 53 years old) "That pick seemed to be for women, I guess, because it’s light… the size and weight were just right… yes, it was light, not heavy" (p2, woman, 49 years old) "The pick is a bit heavy, but there are some that are lighter, that don’t weigh much, right?… My work pick is already light, it’s light… it’s just right for me" (p10, woman, 43 years old) |
|  | Sharpness and maintenance | "Yes, my pick has been less sharp and worn out… you have to take it to a blacksmith to sharpen and straighten it. He sharpens the tip, and that keeps it pointed. You take it whenever you dig, to a blacksmith, and you pay 2.50 soles" (p1, woman 53, years old) "Maintenance is easy. You need, as I say, a bit more wood in that spot to adjust it; if not, maybe at a blacksmith they could make it smaller. (p2, woman 49 years old). "Sometimes, with so much use, it becomes dull (loses its edge), and you have to take it to the man who sharpens it. The blacksmith hammers it… then he sharpens it. It’s just like a knife that’s not sharp; you have to take it there to get it sharpened. And then you can work properly. He charges 5 soles per point" (p5, woman 50 years old) "You just have to sharpen it… take it to get the tip sharpened; to make it sharper… that’s how the blacksmith sharpens it. I take it to the blacksmith to make it finer" (p6, man,47 years old) "We have to sharpen it; it has to be sharp. Sometimes, when you work a lot, the tip wears down, gets small, and you have to lengthen it or buy a new one" (p7, woman,49 years old) |
|  | Components and assembly | "My little allachu is very firm, well attached to the wooden handle" (p3, man 78 years old) "Yes, every now and then the little handle needs a bit of fixing" (p4, man, 55 years old) "We only buy this metal in Abancay… what we do here is use a stick; we get that stick from the field… but some people let it dry, you know, and then we change those little handles" (p10, women, 43 years old) "When we prepare it, we put on a piece of rustic wood from the monte" (p12, man, 47 years old |
|  | Comfort of use | "In the field, it hasn’t moved, we’ve worked normally digging, and we’ve gotten the potatoes out quickly" (p1, woman, 53 years old)  "Yes, it’s uncomfortable, right? It’s kind of loose…" (p6, man, 47 years old) "Nothing has broken; it still works… it’s been fine so far" (p7, woman, 49 years old) "For me, my little pick is fine… at that height, around my waist, it’s good" (p10, woman, 43 years old) |
| Pre-work preparation | Preparation strategies | "You can change the handle, or else reinforce it with a small piece of wood so it’s properly fitted, right? So it won’t be loose anymore" (p2, woman 49 years old) "We have to go well prepared to be able to work; otherwise, how will we get anything done? Of course, it has to be firm; everything has to be ready to work" (p7, woman, 49 years old) "I have to take my tool already prepared, yes, make sure it won’t come off… you also have to consider your strength, like, which pick can you work with? Everything is preparation" (p10, woman, 43 years old) "Before going, you start getting your pick ready, counting how many people are going, and then you figure out what to bring: five picks, three picks, two, like that." (p12, man 47 years old) |
|  | Self-managed maintenance | "Yes, I’m going to change the wood, put in a new little stick… it’s a bit difficult to scrape, it’s like a saw" (p4, man, 55 years old) "We send the replacement to Mr. Julian… it requires the little wedges, the tips; here it comes out, so you put a stick for its wedge" (p8, woman, 41 years old) "You can get the stick here in the monte, bring a stick, and you have to sand it, scrape it… it has to be exact so it won’t come out, you put its little wedge. For the handle, you know, you go to the field to get a stick… you also have to let it dry; you can’t put it in wet" (p10, woman, 43 years old) "When you’re preparing, scraping the wood, since it’s rustic, you scrape it, shaping it to fit your hand. If it’s a little short, you have to scrape some more" (p12, man, 47 years old) |
| Evaluations of the tool | Comfort with their own tool | "My tool is always like this, it hasn’t loosened or come apart […], I work in the garden, the field with my allachu; the tool is good for everything… no, it’s not uncomfortable" (p3, man, 78 years old) "The tip of the pick is always useful, because sometimes we also do bio-gardening" (p7, woman, 49 years old). "I’m just working with this little pick; I also worked with it yesterday, right? It works fine" (p10, woman, 43 years old) |
|  | Displeasure with the available tool | “Yesterday I used another one that was a little heavier, because this one here has a different weight, was uncomfortable” (p4, man, 55 years old) "I was digging like this, chopping, and the potato was already coming out… what do you call it? damaged already, yeah, like I was hitting it, the tip must have hit the potato… and I took it out damaged." (p10, woman, 43 years old) Points to the pickaxe's blade: "Here it’s short, about 20 cm or so… this one here is wide at the back, the middle part is square and wider, just small but wider… on Saturday and Sunday I had to use another tool because there wasn’t another one… it was very wide, very broad, and of course that breaks the potato… is different because you’re used to your own. (p12, man, 47 years old) |
| Consequences of a deficient tool | Inability to work | "Those other days, when it got a bit loose, I felt uncomfortable. Of course, I have to move it forward, I’m working on that, and it makes my hand go numb a little… it makes it slower" (p8, woman, 41 years old) "If you take a bigger, thicker pickaxe that you can’t hold, you're digging and digging, you get more tired. If it breaks… and you’re far away from home, you can’t go back … and if this little pickaxe comes off or breaks, I’ll ask myself, ‘what pickaxe am I going to work with?’ I can’t go back from up there. With this, if the spacing is too small, I can’t work… if it’s too small or too big, I can’t work." (p10, woman, 43 years old) |
|  | Physical risk and additional effort | "Of course, the head was loose, right? It wanted to come off… it’s dangerous and makes my work harder. The waist, when you’re bent over for a long time, hurts" (p2, woman, 49 years old) "I felt discomfort, not just physically from digging so much, but I felt myself bending repeatedly and get injured" (p5, woman, 50 years old) "When you grab it for the first time, it bothers you… the right hand works the most, and your waist is bent, almost the whole day you’re bent over… as I say, I get here all worn out"(p10, woman, 43 years old) |
| Accumulated experience and appropriation | Prolonged use and natural wear | "I was working with that little pickaxe, but it was already loosened. I was struggling (laughs)… it had already loosened, I think, it was making a 'clok clok' sound, and it already needed a wedge."(p5, woman, 50 years old) "Its tip wears out… when it hits a stone" (p8, woman, 41 years old) "From working so much, right? I mean, in two years, one year, it also wears out… the little handle can crack in this part… yes, it also wears out from so much work… sometimes you hit a stone, sometimes the ground is hard, right? by chance you hit a stone, right? And it bends… now it doesn’t have a tip anymore… if it’s bent, it has no strength, no tip, no edge" (p10, woman 43 years old) (About the head) "it’s no longer strong, it’s welded iron… the handles sometimes loosen with use." (p12, man, 47 years old) |
|  | Personal adaptation | "It has to be made your way, according to your judgment… you tell them, ‘I want more,’ you sharpen it more or make it flatter, say, ‘I don’t want this, I want the tip flat,’ and they make it flat, or ‘make it more pointed’… you indicate how you want it" (p6, man,47 years old) Referring to the pick head: "Yes, it also has to have the right shape because sometimes they are bigger. The iron, well, the iron, of course, shouldn’t be too big; it has to be to your liking" (p7, woman, 49 years old) "We prepare it according to our hand… you adapt as you go, since it’s your tool, you inevitably get used to it" (p12, man 47 years old) |
| Family and gender dynamics | Family support | “Yes, (laughs), my husband brings wood from far away (…) *tasta* they call it” (p7, woman, 49 years old) "That’s how the men get used to fixing it; I don’t know how they do it, but they have to scrape the little piece of iron" (p10, woman 43 years old) |
|  | gender roles | "My husband does the maintenance; here almost everyone does their own maintenance, putting on their little piece of iron, their little stick”, “If my husband sees it, he fixes it, right? If it’s a little off, loose, right? When he has time, he starts preparing it… if it’s bad, he says, ‘I’ll get it ready’", "My husband changes the handle" (p10, woman 43 years old) |

Footnote: Individual participants may appear across multiple domains, as the same lived experiences informed different analytical dimensions.
