# Supplementary material for: Andean agriculture and hand tools: A qualitative approach of exploration of needs, barriers, and opportunities for innovation
Source: PLoS One. 2026 May 15;21(5):e0335295. doi: 10.1371/journal.pone.0335295 (PMC13178989; doi:10.1371/journal.pone.0335295)
Supplement: Attachment — Submitted filename: Response to reviewers 1202.doc [file pone.0335295.s009.doc]

**LETTER OF RESPONSE**

Dear editor and reviewers

We would like to thank you for the suggestions and comments made on this manuscript. We are sure of the improvement of this based on your recommendations.

Below you will find the answer to each point.

Kind regards.

**Journal requirements:**

1. Please ensure that your manuscript meets PLOS ONE's style requirements, including those for file naming. The PLOS ONE style templates can be found at

<https://journals.plos.org/plosone/s/file?id=wjVg/PLOSOne_formatting_sample_main_body.pdf> and<https://journals.plos.org/plosone/s/file?id=ba62/PLOSOne_formatting_sample_title_authors_affiliations.pdf>

**Response**: The manuscript has been revised to adjust PLOS ONE´s style requirements.

2. Please include a complete copy of PLOS’ questionnaire on inclusivity in global research in your revised manuscript. Our policy for research in this area aims to improve transparency in the reporting of research performed outside of researchers’ own country or community. The policy applies to researchers who have travelled to a different country to conduct research, research with Indigenous populations or their lands, and research on cultural artefacts. The questionnaire can also be requested at the journal’s discretion for any other submissions, even if these conditions are not met.  Please find more information on the policy and a link to download a blank copy of the questionnaire here: <https://journals.plos.org/plosone/s/best-practices-in-research-reporting>. Please upload a completed version of your questionnaire as Supporting Information when you resubmit your manuscript.

**Response:** The inclusivity in global research is added as a Supplemental file-S3

3. Thank you for stating the following in the Acknowledgments Section of your manuscript:

“This work was funded by the Consejo Nacional de Ciencia, Tecnología e Innovación Tecnológica (CONCYTEC) and the Programa Nacional de Investigación Científica y Estudios Avanzados (PROCIENCIA) within the framework of the E077-2023-01-BM “Becas en Programas de Doctorado en Alianzas Interinstitucionales” contest, grant number (PE501090201-2024-PROCIENCIA-BM) and the E033-2023-01-BM “Alianzas Interinstitucionales para Programas de Doctorado”, grant number N° PE5010843066-2023-PROCIENCIA-BM).

Please remove any funding-related text from the manuscript and let us know how you would like to update your Funding Statement.

**Response:** Thank you for the clarification. The funding statement states as follows:

This work was funded by the Consejo Nacional de Ciencia, Tecnología e Innovación Tecnológica (CONCYTEC) and the Programa Nacional de Investigación Científica y Estudios Avanzados (PROCIENCIA), within the framework of the E077-2023-01-BM “Becas en Programas de Doctorado en Alianzas Interinstitucionales” contest, grant number N° PE501090201-2024-PROCIENCIA-BM and the E033-2023-01-BM “Alianzas Interinstitucionales para Programas de Doctorado”, grant number N° PE5010843066-2023-PROCIENCIA-BM. The sponsor did not play any role in the study design, data collection and analysis, publication decision, or manuscript preparation”

4. We note that you have indicated that there are restrictions on data sharing for this study. PLOS only allows data to be available upon request if there are legal or ethical restrictions on sharing data publicly. For more information on unacceptable data access restrictions, please see http://journals.plos.org/plosone/s/data-availability#loc-unacceptable-data-access-restrictions.

**Response:** The de-anonymized dataset has been uploaded to the OSF repository and is available at: <https://osf.io/fvr9t>

**Reviewer’s comments**

***Reviewer #1:***

1. *Abstract: Concise and well-formed*

*Introduction: well written*

*Material and methods: nicely explained, but no scientific approach is used to collect data.*

*Results****:*** *Tables and figures are elaborated very well. Each head was clearly explained. The statements made by participants clearly depict the existing situation of respondents by using hand tools. It is a detailed qualitative approach for analyzing hand tools. However, In the introduction, health hazards and MSDs caused by the use of poorly designed hand tools were discussed. But in the study, the ergonomic points of the tools' analysis were missing. Since no statistical tool has been employed to infer the findings, the results do not appear to be statistically sound. Health risks and MSDs brought on by using a hand tool with inadequate design were covered in the introduction. However, in the study, ergonomic tool analysis is absent Second, the results are not inferred using any statistical tools. As a result, the study's overall findings are lacking. The result should focus on some clear and quantifiable information, like which parts of the hand tool were deemed to have poor ergonomic design or require revision. In the results, dimensions of hand tools are missing, which are necessary for assessing the tool, such as weight, length, and size. It is not ergonomically sound to analyze tools just based on statements*

**Response**: Thank you for the comments. We strengthened the methodology and results section of the manuscript by complementing the qualitative findings with descriptive quantitative synthesis. In the results section, pain-related codes were quantified by body region at the participant level and are now reported as the number and percentage of participants who mentioned pain in each region at least once (presence/absence per interview), using a binarized code–document table in ATLAS.ti v25. These results are now presented in Table 1 (n/N, %), including stratification by sex. In addition, we incorporated an ergonomic description of the hand tools assessed, reporting their physical/ergonomic characteristics in Table 2. And a summary of tool components/parts reported by farmers as problematic. Quotations were retained only as illustrative examples; the interpretation and discussion are now grounded on the quantified results and tool measurements. It is worth mentioning that a more comprehensive ergonomic assessment (anthropometry, observational evaluation such as REBA, biomechanical analysis, and sEMG) is addressed in a separate manuscript; however, this paper remains focused on farmers’ experiences while incorporating the above descriptive quantitative summaries and tool characteristics to support and contextualize the findings.

2. *Further, both men and women were included in the study as samples; however, they differ greatly in their physical characteristics and how they use hand tools.*

**Response**: We agree. Men and women can differ in physical characteristics (anthropometry, height, etc.) and in how they use hand tools, which may affect discomfort and tool suitability. We updated the manuscript to present the MSDs outcomes (pain and discomfort) stratified by sex and to interpret these differences in relation to the measured physical/ergonomic characteristics of the tools. Also, we added an anthropometric measurements table in supplementary file 5, to complement and better understand physical characteristics differences among participants. Lines 285-294.

3. *Information added: Farmer-provided or farmer-made sketches for modifying the current tool design are unclear. All recommendations for creating or making changes to the tool design can be included in a sketch or drawing (in CAD). It will be a meaningful finding and feedback for further investigation.*

**Response**: We appreciate the suggestion. The sketches produced by the farmers were included as originally made in the Supplementary Material because our aim is to faithfully preserve the feedback generated during the participatory process. These drawings serve as qualitative inputs to identify opportunities for improvement (e.g., adding a cutting edge to the blade/tip, adjustments to weight, length etc.), rather than as proposals with defined dimensions and geometry. For this reason, we did not convert them into CAD in this manuscript. Producing CAD models would require assuming measurements and technical details that are neither contained nor validated by the original sketches and could therefore introduce an artificial level of precision. The translation of these suggestions is undertaken later during the detailed design stage.

4. *Discussion: Comprehensively written by detailing each component. But the discussion is only based on statements of farmers, not on the crux of the results. A statement made by one farmer does not reflect the opinion/point of view of other farmers. So, there should be some quantification of data/results to interpret the findings in a meaningful manner.*

**Response:** We agree with this observation. To ensure the discussion is grounded in the core results and not only on individual farmers’ statements, but we also incorporated a quantitative summary of the findings. Specifically, we added results on bodily discomfort and pain reported by farmers stratified by sex, and we present these outcomes in relation to the physical and ergonomic characteristics of the hand tools used. This allows the discussion to reflect patterns across the sample and supports a more meaningful interpretation. Lines 539-570

5. *Conclusion: Not explaining the exact picture of the result. It should be rewritten to highlight the major findings of the study.*

**Response**: Thank you for the suggestion. We rewrote the conclusion section to include relevant findings. Lines 661-677

6. *Supporting information: The detailing of each component is added and clearly exhibits the research.*

**Response**: Thank you

***Reviewer #2****.*

1. *The paper is a quite focused study of farmer attitudes and perceptions regarding their hand tools in a Peruvian Andean community. It is a bit reduced in terms of overall scope and sample size, the latter of which the authors acknowledge. It would have been nice to do the study across a number of communities with a smaller set of farmers in each community, to increase the generalizable nature of the results. However, as this is a relatively novel aspect of a more general occupational health/literature, there is something to be learned here. My two main critiques of the paper and reason for major revisions are first, that the methods and especially the survey guides need to be more clearly explained in terms of their objectives, link to the research questions, differentiation among the two, and description of the areas covered or the technique. Sometimes the objective of a guide is only found in the results, very late for building up the understanding of the methodology. Without this, the thematic areas of the results seem rather haphazard.*

**Response:** Thank you for these comments. We agree that conducting the study across multiple communities would have strengthened generalizability; however, this was not feasible due to limited financial and human resources, and we now state this more explicitly as a study limitation. Regarding the methodology, we revised the Methods to clarify the objectives and scope of each guide, their alignment with the two research questions, and the differences between guide 1 and 2. We strengthen the Data analysis section to make clear how the results were organized: coding was guided by a priori analytic domains (human, product, task, qualitative), with codes developed inductively from the data; an additional environmental domain emerged during coding and was incorporated as a fifth domain. Finally, we state that results are reported according to these five analytic domains. Lines 109-129; 168-180

2. *Second, within the limitations of a small and potentially overly ‘unique’ (i.e. one village) sample size which is already acknowledged, I would urge the authors to expand a bit on the wider meaning and implications of the results. What about the observation that manufactured tools are increasingly prevalent and older, bespoke tools are increasingly rare? Are there implications from increasing mechanization and demographic change? In general, there is a need for a bit more detail in certain areas of the paper where the logical flow or background to information is missing, as is noted below in the detailed line by line comments.*

**Response:** Thank you for the comment. We have expanded the Discussion section to improve the logical flow and to situate our results within wider demographic and technological changes occurring in Andean agricultural context. Drawing on field observations, we added a paragraph describing how rural out-migration of younger populations, the aging agricultural workforce, and the availability of imported tools and replacement parts shape farmers’ decisions. Lines 626-638

3. *Also, for ease of reviewing if I review this manuscript again, I would ask that the figures be placed within the text, it makes it much easier and actually increases the speed and quality of the review.*

***Response****:* Dear reviewer Initially, we included the images within the manuscript as requested; however, according to the PLOS editorial policy, images must be submitted as separate files, which is why we could not finally include them within the manuscript.

4. *One last point, I have indicated that I don’t feel this paper meets the data sharing standards of PLOS One. That is because individual access with consent of the authors to the data is not really essential in my view. It should be possible to anonymize the source testimony material and erase any personal mentions such that the database would remain as a textual resource showing in a more detailed way all the attitudes towards tools and their context. If the ethics board is denying this option then the authors may claim so, and perhaps the main editor will disagree, but I would urge you just to prepare a text resource in this way, with no personal or geographic information, and share, even in the online materials. That is very broadly in line with ethics standards, especially as these are opinions about management and tools rather than personal, health, or family details.*

**Response**: Thank you for this recommendation. In line with Plos One data sharing standard, we have prepared a de-identified textual dataset derived from the source testimony material. All direct identifiers and potentially identifying details (e.g., names, personal references) were removed to minimize re-identification. Data is available at: <https://osf.io/fvr9t>

***Detailed comments with line numbers:***

*47 ecological variability not variety*

**Response:** text corrected. Line 48

*55 Looking at your references here, I think that you should approach the statement “many of which lack ergonomic principles” with more skepticism. I am wondering whether you are conflating lack of ergonomic principles with lack of mechanization which could lead to less overall effort and fatigue? At least in some of the Andean contexts where I have worked, and in fact as you document in this paper, farmers indicate strong preference for certain of their tools and even will approach blacksmiths with particular design elements that make tools more suited to their work setting. However, this may be different in Peruvian Andean contexts if all tools are increasingly manufactured outside (e.g. Brazil, China) and imported to the region – but perhaps separating effort, fatigue, and efficiency is important. you may be able to use the literature on hand tool use from beyond the region that you already cite, that will speak to this point?*

**Response:** Thank you for the observation. We revised the text to clarify that Ergonomic adequacy should not be equated with the absence of mechanization, but rather the design of work systems, including tools. We emphasize that manual tools may generate musculoskeletal risk when tool design and task demands are not aligned with human capabilities, even when such tools are locally preferred. In addition, we incorporated evidence from hand tool use in non-agricultural settings to support this distinction. The revisions are included in lines 56-63

*62 This is a very generic reference to a textbook (Harrington 2000, Occupational Health) for the notion that tool redesign could help farmers --- could you provide a more specific reference focused on farm environments, or examples of tool design that id in fact help farmers, if you mean to link this to literature?*

**Response:** We thank the reviewer for this comment. The generic textbook reference has been replaced with more specific agricultural ergonomics literature. We now cite intervention-based studies conducted in farm environments supporting the idea that ergonomic and anthropometric redesigning traditional agricultural tools can reduce physiological workload and improve task performance**.** Lines 65-72

*67 I would say “ innovating further on technological solutions “ rather than “Developing”--- the hand tools analysed are in fact already a technological solution, sometimes developed by farmers, and you are making the point here that their context and knowledge should be respected!*

**Response:** Thank you; this has been clarified and incorporated into the text. Line 84.

*71-72 more details on this design model? It’s not commonly known, e.g. what do the “two diamonds” refer to? An example of more detail being needed to fully understand your objectives – a few sentences*

**Response:** We have added a brief explanation of the “Double Diamond” innovation framework, clarifying what the “two diamonds” represent and how the stages relate to our approach. We also included an example of its application in an agricultural context to better illustrate the framework and strengthen the rationale in the introduction. Lines: 88-94

*75 are these the two objectives of the two instruments below in methods? Do a better job identifying how the methods address these two main questions, then. Also, I am thinking that the first question is more general around needs and context, and the second is more specific around their specific reactions to a set of tools in harvesting. If that is the case, use that terminology (general/context vs. specific etc.) to aid the reader’s understanding.*

**Response***:* We appreciate the comment. We have clarified the scope of both research questions (contextual vs. specific) and, in the Materials and Methods section, we have explicitly stated how each interview guide addresses each question. Lines 95-102, 109-129

*77 I think you need defend the choice of one particular operation of potato harvesting. Why choose that compared to say, carrot peeling or compost pile turning – tools are used for all of these? I am assuming the choice of potato harvesting was not just opportunistic. It's a pretty central focus of agriculture in Peru etc.*

**Response:** We have revised the manuscript to clarify that this choice was intentional rather than opportunistic. The text now explains that potato is Peru’s main temporary crop, representing 41.3% of national temporary crop production in 2022, and is cultivated extensively across the Andean highlands as well as in central and southern coastal regions, representing a central role in national food security. In addition, potato harvesting relies heavily on manual labor and concentrates multiple ergonomic demands. Lines 73-79

*83 – you mention two guides but don’t say how they differed, what was the purpose of each, why two? - if that is the meaning of “and” later in the sentence, enumerate these as the two purposes. More detail and clarity needed.*

**Response:** Thank you**.** We clarify the purpose of each guide in relation to the stated question in the section: Interview guides: objectives and alignment with research questions (Material and methods) Lines: 113-129

*142 “Data are presented according to the interview guide used” but I don’t understand the difference between the two interview guides, as the methods are incomplete, and here just below you say the first interview guide described perceptions, but again it’s not clear and doesn’t distinguish this from the second guide.*

**Response:** We thank the observation. We have revised the Methods section to provide a detailed description of each guide and to clearly distinguish their objectives and analytical focus. The manuscript now explains that two semi-structured interview guides were used, each aligned with one of the study’s research questions. The first guide explored farmers’ perceptions, needs, and contextual factors related to manual tool design and adoption, while the second focused on post-harvest experiences, perceived performance, and satisfaction with traditional tools. We also clarify the timing of application, content areas covered. Lines:99-102, 113-129.

*151 Task category – is this a structure from the interview guide, or an emergent theme from coding?—this all needs to be presented more clearly. Perhaps linking to the figures with the different guide numbers to a greater degree within the text will help?*

**Response:** Thank you. We have clarified in the Data Analysis section that the “task” category was defined a priori as part of the analytical framework derived from the human–hand tool system proposed by Jain et al. and did not originate from the structure of the interview guides. Lines 168-180

*184 adapt as in choosing different alternatives, or adapt as in modifying their tools? Two different things. Again, the language here is not very specific and seems to lack the complexity that is promised in the introduction.*

**Response:** Thank you for the observation. We revised the text to clarify the meaning of adaptation. In this context it is referred to adjusting pre-existing hand tools through physical modification, reflecting the different strategies farmers use in practice. Lines 244-247

*194 here I would say just children, without the definite article The – we are not talking about particular children.*

**Response***:* Thank you for the observation. The term has been corrected. Line 253

*196 Is this really true that there are no individual plots? Is this a communal sectoral fallow system with use rights granted by a communal entity like a consejo? Because in many areas of Peru labor exchanges may be practiced (some labor is thought of in a common way) but in fact the plots are held privately by families. It would be good to clarify this so that you are not propagating a vague or romantic image of communities holding land in common, if it is not the case, to complement what the farmer said. Maybe clarify this using a comment on this quotation*

**Response:** We added a brief interpretive clarification following the participant quotation to distinguish collective labor practices from land tenure arrangements. In the study area, as in many regions of the Peruvian highlands, land-tenure arrangements coexist. Most resident families cultivate parcels that are inherited and socially recognized within the community, often under the oversight of communal authorities. At the same time, a smaller proportion of households do not hold individual land titles and participates in rotating or shared-use parcels. We have revised the manuscript to clarify this distinction and to avoid portraying land tenure as uniformly communal or idealized. Lines 258-265

*221 it is always 7 days, or just as reported by the participants in this study? – or that this is somehow important to the particular focus on the design of hand tools? I find it hard to believe that some other factors like weather or yield levels might not make it last more or less time? In general this seems to reveal a weakness of doing a study in one community in one year, it needs to be carefully presented to not overgeneralize, and it would have been nice to have participants from a range of communities instead of just one, seems like pseudoreplication of sorts.*

**Response:** We revised the manuscript to clarify that the reported seven-day harvesting period reflects participant accounts from the study community during the study year and does not represent a fixed or general duration. The wording was adjusted to avoid overgeneralization. Limitations related to the single-community design were also acknowledged in strengths and limitation section. Line 298-300

*231 What should resemble the pick? Use parentheses to identify the referent.*

**Response***:* We clarify this. So it refers to the pick head. Line 311.

*247 this actually parallels what I observed in Bolivia in the 2000s, both allachu (called wiluku, there) and picks were used. Given the emphasis on physical lightness of the tools, did no one comment on the fact that a pic k is more versatile with two sides but in fact obligates one to carry the weight of a function that is not always used, versus having more or less pointed allachus that are lighter and perhaps more adapted to to the main task of potato harvesting? Also, is the soil in this community rocky, so that extracting or moving rocks is such an important task?*

**Response:** Thank you for the comment. We´d like to mention that farmers do not seek minimal weight alone, but rather a balance between weight and effectiveness. Participants described that heavy tools increase fatigue, whereas overly light tools are ineffective for breaking the soil. Moreover, the preference for dual-ended tools reflects their functional complementarity: the pointed tip is used to penetrate and fracture the soil, while the flat blade facilitates soil removal and potato exposure. In the study area, soils are predominantly clay-based, and although large stones are uncommon, compacted soil clods frequently form during harvesting need to be broken to release the crop under these conditions, the combined use of both ends was perceived as advantageous. We added this trade-off between tool lightness and multifunction in discussion section. Lines 539-549

*248 is the prevalence of blacksmiths decreasing or staying the same? Did you ask about this? It seems like if blacksmithing skills are lost this would be a natural explanation for the transition to different pick tools that come from outside, and this could be an implication of the study, a need to support more local tradespeople or accomplish that function within local agencies or development organizations?*

**Response***:* During fieldwork, participants explained that blacksmithing is not practiced as a full-time occupation within the community. Repair and tool-adjustment activities are instead carried out by local farmers who acquired these skills empirically and provide services upon request. For these individuals, blacksmithing represents a secondary activity, with agriculture remaining their primary occupation. Participants differentiated clearly between tool components. The metal head was considered critical for performance and safety and, when damaged or requiring reshaping, was usually taken to a local blacksmith. In contrast, wooden handles were more frequently adjusted or replaced by farmers themselves, using locally available materials or commercially purchased replacement handles from local hardware stores, which are typically imported.

One participant reported performing occasional blacksmithing work at home using basic tools and experiential knowledge rather than within a formal workshop. No additional full-time blacksmiths were identified through participant accounts, although this aspect was not systematically explored.

We hypothesized that access to metal repairs depends on the availability of these individuals, which may be limited during peak agricultural periods such as harvesting. In comparison, replacement handles can be obtained immediately from hardware stores, making self-replacement a faster and more accessible option.

We added this reflection to the discussion section. Lines 596-604

*275 “3.0+/- 0.2 kg” is the +/- a standard error, standard deviation range mentioned by interviews, what? Please note quickly*

**Response:** We clarified that 3.0 ± 0.2 kg is reported as mean ± SD (and added n), to avoid ambiguity. Line 355-356

*281 I think that what p5 is describing here is a sort of local optimum of weight, neither too light or too heavy, as you also say in lines 343 – maybe these results on intermediate weight and relation to age and gender could be consolidated.*

**Response:** We revised the Results section to clarify the analytical distinction between the product and qualitative domains. Weight is now presented in the product domain as a physical attribute influencing adoption and replacement decisions, while in the qualitative domain it is addressed as an embodied in-use experience shaping efficiency, fatigue, and comfort. We also consolidated these findings by highlighting an intermediate optimal tool weight that varies according to individual characteristics such as age and gender. Lines: 354-357, 444-452

*317 this is the same quote as in the previous section that presumably comes from the first interview guide and here the second? It seems a bit repetitive as it makes the same point.*

**Response:** Thank you for noting this repetition. The quotation indeed originated from the first interview guide and was inadvertently repeated in the subsequent section. To avoid redundancy, the duplication quote has been removed, and the section has been revised. Lines 418-424

*332 only now you describe what the second interview guide is about! All this should be described clearly in the methods section.*

**Response**: Thank you for this observation. We have revised the manuscript to clearly describe the purpose and scope of each interview guide in the Methods section. The objectives of both guides are now explicitly stated to clarify their respective roles in data collection and analysis. Lines 123-129

*430 Please change to Jain et al.’s (2018) proposal for improving…. To clarify that this is a reference to an existing paper. Also Jain is not a single author, etc.*

**Response:** Thank you, we corrected the cite. Line 536

*431 rephrase as “While adding environment as a new theme within their narratives” or similar. The colon is awkward here since only two words follow it. At a minimum you can say “the environmental theme” (and also refer to the figure 5 again with these themes)*

**Response:** Thank you, we corrected the text. Lines 538

*446 by multiple heads please clarify whether you mean a tool with two heads (or more?) or just multiple tools with different sized heads to adapt in the field to the soil conditions.*

**Response:** Thank you. To clarify this, we revised the text to indicate that farmers expressed preference for interchangeable tools heads. Lines 572-575

*453 I don’t know that you need a reference to explain the connection between humidity, rain and rotting of wood. Better to stay focused on the farmer viewpoints, rotting and weakening is a well known process.*

**Response:** Following this comment, we removed the unnecessary reference and revised the paragraph to focus on farmers’ viewpoints.

*462 Can you specifically point to where in your research or in which aspects farmers identified the tradeoff between productivity and comfort? I am not remembering a farmer talking about this and since it is a bit contentious, would like to have the connection to the testimonies clearly described. Would not an uncomfortable tool fatigue a farmer more and thus lead to a shortened and thus less productive work day? So it needs to be clearly explained.*

**Response:** Thank you for this clarification. We agree that the trade-off between comfort and productivity was not explicitly articulated by participants in the interviews. We revised the manuscript to clarify that this point is presented as an interpretative reflection grounded in the literature, rather than as a direct empirical finding from this study. This reflection helps to contextualize potential barriers to the adoption of ergonomically improved tools beyond issues of familiarity or similarity to traditional implements. Lines 587-592

*478 instead of substitutes would say “acts in place of” or “substitutes for” , I slightly prefer the former because it is somewhat more neutral, not privileging formal or written learning pathways.*

**Response:** Thank you, we corrected the term. Line 615

*481 although you seek to correct a bias in favor of formal versus informal intergenerational training in the next sentences, you could strengthen that perspective in this line by saying “ but rather from the absence of training that would accompany an outside tool in a more formalized technology context” ? because I think the formal training route only applies to tools or methods that are brought from outside? (Whether or not they are effective; sometimes outside tools and formal training can be effective, but as you point out local pragmatic learning enhances a sense of ownership of the tool technology)*

**Response:** We agree with that and revised the manuscript to explicitly distinguish between locally transmitted, intergenerational learning and the lack of accompanying training when externally developed tools are introduced. Lines 614-623

*492 no capital C on characteristics.*

**Response:** Corrected. Line 642.

*502 I’m glad you recognize the sample within one community as a limitation, I agree – better to document this limitation for readers than ignore it.*

**Response:** Thank you.

*512-515 You mention the way that a local repair function is an enabler but I think maybe this should be included in the last few lines that are the main prescriptive outcomes of the paper – that is, whether tools are made locally or come from outside, they will be much more valuable in communities like this one if they are repairable and modifiable. As I noted regarding the results section, it might also flag the availability of skilled blacksmiths in these communities or near to them, as a key factor in the sustainability of hand tool use that should be explored in addition to the other factors mentioned.*

**Response:** Thank you for this suggestion. We agree that local repairability is not only an enabling factor but also a key prescriptive implication of the study. We have revised the Discussion to integrate this point into the concluding, forward-looking section of the manuscript.

Specifically, we now emphasize that hand tools, whether locally produced or externally manufactured, are more likely to be sustainable and valued in communities such as this one when they are repairable and modifiable at the local level. We also highlight the availability of skilled local blacksmiths, even when blacksmiths are a secondary occupation, as an important factor supporting ongoing tool adaptation and maintenance. Lines 642-646.
