# Supplementary material for: Andean agriculture and hand tools: A qualitative approach of exploration of needs, barriers, and opportunities for innovation
Source: PLoS One. 2026 May 15;21(5):e0335295. doi: 10.1371/journal.pone.0335295 (PMC13178989; doi:10.1371/journal.pone.0335295)
Supplement: Attachment — Submitted filename: Response to reviewers.docx [file pone.0335295.s010.docx]

**LETTER OF RESPONSE**

Dear editor and reviewers

We would like to thank you for the suggestions and comments made on this manuscript. We are sure of the improvement of this based on your recommendations.

Below you will find the answer to each point.

Kind regards.

**Reviewer´s comments**

*Reviewer #1:*

1. *Abstract: Well-structured and provides a clear overview of the objectives, methodology, major findings, and significance of the study. It effectively summarizes the core aspects of the research and reflects the essence of the manuscript.*

*Introduction: clearly establishes the importance and relevance of the topic. The background information is well articulated and supported by appropriate and relevant literature. The cited studies help in establishing the research gap and justify the need for the present investigation.*

*Materials and methods: This section is now systematically organized and clearly presented. The headings and subheadings effectively describe the procedures and methodological approaches adopted for data collection and analysis. The section is sufficiently detailed to allow readers to understand the research design and the processes followed in conducting the study.*

*Results: The results section reflect the considerable effort made by the researcher to present the findings comprehensively. The major recommendations provided in the earlier review appear to have been incorporated. The findings are well organized and presented in a technically sound manner.*

*Discussion: The researcher has made a commendable effort to interpret the results and explain the underlying reasons for the observed findings. The discussion appropriately relates the results to the perceptions of respondents regarding agricultural work and the use and application of hand tools in their daily work practices.*

*Conclusion: Concise and clearly highlights the major findings derived from the study.*

*Overall, the researcher has made significant efforts to improve the manuscript. The revisions have strengthened the quality and clarity of the paper. In its present form, the manuscript represents a meaningful contribution to the field and will be useful for future researchers working in this domain.*

*Recommendation*

*Based on the improvements made and the overall quality of the manuscript, the paper is recommended for publication.*

**Response:** Thank you for the comments.

Reviewer #2:

*I am pleased to say that the authors have addressed the questions in my review and have substantially improved the manuscript. I am only making some detailed suggestions for editing that should be considered to remove errors and make the manuscript more readable. These should take very little time and I then endorse the manuscript for publication.*

*Detailed comments*

1. *35 would suggest inserting here “We found that hand tools used in…” to delineate this section of the abstract as results*

**Response:** Corrected. Line 35-36

2. *44 what are MeSH terms? Maybe spell out or just write them?*

**Response:** Thank you for the comment. MeSH (Medical Subject Headings) terms are standardized keywords used in biomedical databases to index and organize articles. The term has been spelled out. Line 44.

3. *49 It may not be germane to the research topic, but especially if you are setting the bar at 97% of farms, I don’t think these are exclusively subsistence systems. As you clarify below, some of their crops are for sale presumably if 66% is for home consumption? It’s not crucial for your study but I would find a different term, perhaps diversified smallholder farming (because income streams are increasingly diverse, not just farming, mining etc.). It doesn’t undermine any of your design or findings, not a dealbreaker so to speak, but is just a chance not to perpetuate myths about Andean farmers.*

**Response:** Thank you for the observation. We agree that the term “subsistence family farming” may not fully capture the complexity of these farming systems. Accordingly, we have revised the text to use “diversified smallholder family farming,” which better reflects the combination of self-consumption and market production, as well as the diversity of income sources among these households. Lines 49-50, 54.

4. *52 allocate, the previous sentences are in present tense as is appropriate and here past tense is appearing. Please check tenses throughout; intro generally present, methods, results narrated as past; and discussion probably a mix of present and past…I think I found most of the instances of mis-tensed verbs but a check would be good.*

**Response:** Thank you for the comment, we checked the tense time use throughout the manuscript

5. *69 tools*

**Response:** Corrected. Line 66.

6. *72 period at end of sentence*

**Response:** Corrected. Line 72

7. *74,75, etc. temporary, I would say annual. Temporary does not have that implication in English; annual vs. perennial crops is the usual distinction.*

**Response:** Thank you for this clarification. We corrected the term. Line 74

8. *76 I think you are missing a word, national importance or prevalence (to not be redundant with importance below)?*

**Response:** Thank you for the observation. We added the missing word “relevance”. Line 76.

9. *78 I would say important context rather than particular*

**Response:** Thank you for the observation. We corrected the term to clarify the idea. Line 78

10. *88 More of a transition is needed, e.g. “To address this need for co-creation, the “double diamond” design model… otherwise too sudden.*

**Response:** Thank you for this comment, we corrected the phrase to improve transition. Line 88.

11. *93 define not defines*

**Response:** Corrected. Line 93.

12. *97 I am not sure that the text after the colon is needed, … two questions: 1….2… is sufficiently clear. Was “a contextual question (needs…) “ extra text left after editing? Because it is quite confusing – reword or omit.*

**Response:** Thank you for the comment. We have removed the additional text after the colon, as it was unnecessary and caused confusion.

13. *113 (same for line 123) Less awkward is simply “Guide 1 addressed research question one…” (past tense needed)*

**Response:** Corrected. Lines 113, 123

14. *120 put (Q18-19; see supplemental file one) in parentheses at end rather than as a sentence, or “For more details, see supplemental file 1”. more readable.*

**Response:** Thank you for this suggestion. We have revised the text by placing the reference to the supplemental file in parentheses at the end of the sentence to improve readability. Line 120.

15. *129 Similar to above, parentheses can be combined as (Q6; supplemental file 1)*

**Response:** Corrected. Line 129.

16. *171 Jain et al. works – I would rewrite this slightly, probably “works by Jain et al. (e.g. Jain et al., 2018)” – if I see correctly in your discussion the other Jain reference, with at least one key reference for the reader.*

**Response:** Thank you for this suggestion. We have revised the text to “works by Jain et al.” and included a representative reference (Jain et al., 2018). Line 171,172.

17. *188 slight rephrasing ...emerged during analysis (in this case, after the 11th interview).*

**Response:** Thank you for the suggestion. We rephrased the sentence. Line 188,189

18. *190 is it not ‘anonymized data?’ de-anonymized would be with the names put back in, not what is wanted!*

**Response:** Thank you for this observation. We corrected the term to: “anonymized data”. Line 190.

19. *197 Would just shorten to “ ..is included in Supplemental file 3.”*

**Response:** Corrected. Line 197.

20. *218 I am not sure it is in the pdf but please check spaces in e.g. Fig. 2, Fig. 3 – should have a space between fig. and number.*

**Response:** We checked the documented and corrected the spaces.

21. *257 perhaps better written with a comma, unless I am understanding the farmer wrong: “…is collective, is a communal workday” this reflects better the speech pattern, for me.*

**Response:** We added a comma to clarify the speech pattern. Line 256.

22. *260 If they are regulated through titles I think the word in English would be ‘private’ rather than inherent; or “owned” ? Inherent does not have that meaning.*

**Response:** We agree that, so we changed the term to “owned”. Line 259.

23. *273 could remind the reader of the meaning of allachu here with allachu (hoe) and maybe even (hoe; Fig. 4).*

**Response:** Thank you for this suggestion. We have clarified the term by adding “allachu (hoe; Fig. 4)” to remind the reader of its meaning. Line 272.

24. *288 I would write simply “…three of 13 participants” in the text (no “each”, or I am somehow not understanding what you mean by each); the table fractions are well understood.*

**Response:** Corrected. Line 287.

25. *326 I think tasta and chachas are woods? Maybe say (made from tasta or chachas wood) to complete the thought.*

**Response:** That´s correct. We added “wood” to complete the thought. Line 326.

26. *334 did the farmer use the singular? They did not say ‘allachus’ ? just checking.*

**Response:** that’s correct, the farmer refers to allachu in singular.

27. *353 (barriers and enablers) no capital B*

**Response:** Corrected. Line 354.

28. *368 Which diameter? That of the handle? And this was measured where the hand engages it? Below you use the term grip diameter which is very clear, probably say that?*

**Response:** Thank you for this comment. We have clarified the terminology to avoid confusion. “Handle diameter” is used to refer to the tool measurement, whereas “grip diameter” refers to the user’s hand measurement. Accordingly, we specify “the average handle diameter of both tools…” and, in the following lines, “the maximum grip diameter for women was…”. This distinction helps to clearly differentiate between both terms. Line 369.

29. *Table 2 – is circumference of grip redundant with diameter of grip (handle), ie. Just multiplied by 3.14156…? if so probably only one is needed, to simplify the table, I would just cite the diameter.*

**Response:** Thank you for this observation. While circumference grip and handle diameter are not identical measures, they are directly related and therefore convey overlapping information. To simplify the table and avoid redundancy, we have removed the circumference grip measure and retained only the handle diameter.

30. *387 you may want to shorten this title somewhat, it usually should not run to two lines in the journal, eg. After the colon: “..weight, size, design and other material characteristics” ?*

**Response:** Thank you for this suggestion. We have shortened the title to improve readability while preserving the key elements. Now it says: “product consideration: weight, size, handle, material characteristics and durability”. Line 388,389.

31. *Table 3 this is a nice addition, gives some very specific design considerations. I wonder whether it is necessary to give the /13 or /7 e.g. in the columns, probably just giving the absolute and percentage is enough, since you give the denominator in the heading. And, in the heading you could just say “ out of 13” e.g.*

**Response:** Thank you for this suggestion. We have revised Table 3 by removing the denominators from the cells and indicating the sample size in the column headings.

32. *415 (side topic) I would be interested in knowing if, assuming tools are put away in a relatively dry place, whether it is rust or the physical wear in the soil/rocks (which is definitely apparent in older tools I have seen) that is most important (e.g. for you, line 458). A worked tool quickly regains its shine in the soil eg.*

**Response:** Thank you for this interesting observation. In our field data, participants primarily referred to climatic factors such as humidity and rainfall as contributors to tool deterioration. However, we agree that mechanical wear resulting from repeated contact with soil and rocks is likely to play a significant role, particularly under intensive use. While this aspect did not emerge explicitly in participants’ narratives, we have incorporated a brief reflection in the discussion to acknowledge that tool degradation may result from both environmental exposure and abrasive interactions during use.

33. *416 Are we switching to the second research question and interview guide now? Would be good to flag that! You say that in line 437, but does it actually begin here and deserves noting?*

**Response:** Thank you for this observation. The transition to the second research question is introduced later in the text. To avoid confusion, we have made this transition more explicit by clearly indicating the shift to the second research question and interview guide.

Now it says: “The following section addressed the second research question, aimed at understanding the perceived satisfaction of farmers regarding the use of traditional hand tools. The resulting categories and codes are presented in Fig. 5, and additional representative quotes are presented in Supplemental file 7”. Lines 438-441.

34. *446 would delete however, redundant with while*

**Response:** Corrected. Line 447.

35. *447 associated if we are still in results (past tense, not general knowledge)*

**Response:** Corrected. Line 449.

36. *474 varied, included, -- keep the results in past tense as you are doing throughout most of these paragraphs.*

**Response:** Thank you for this observation. We have revised the verb tense in the Results section to ensure consistency and maintain the use of past tense throughout.

37. *489 Was this something that became apparent in the fieldwork (Evaluation as the core of satisfaction?) or a general statement coming from your research framework? If the former, maybe “Evaluation emerged in the field interviews as core to … “*

**Response:** Thank you for this observation. This aspect emerged from fieldwork. We have revised the text to clarify that evaluation was identified through participants’ accounts as a core aspect of satisfaction. Now it says: “Evaluation emerged in the field interviews as a core aspect of farmers’ satisfaction, encompassing both functional performance and the comfort/displeasure using hand tools…” Line 491,492.

38. *491 I don’t quite understand the phrase “though not all are fully accepted” – the assessments? The expectations? Implicit tool criteria? Please reword for clarity.*

**Response:** Thank you for this observation. We have reworded the sentence to clarify that tool acceptance was not uniform and depended on specific design features, such as the shape and width of the tip, which influenced the ease of use. Now it says: “Positive assessments arose when tools met user’s expectations and working conditions. However, tools were not uniformly accepted, as specific design features appeared to influence the performance and ease of use”. Lines 493-495.

39. *515 I think you mean “Were inherent”? but in any case the phrase “experiences were inherent to agricultural activity but also became more familiar” is unclear to me, as regards both literal meaning and significance? Can you rephrase? Maybe stressing that the familiarity of the tool is an important component as the quotes seem to suggest?*

**Response:** Thank you for this observation. We have rephrased the sentence to improve clarity and emphasize that familiarity with the tool, developed through repeated use. Now it says: “This category encompassed farmers’ narratives regarding the prolonged use of their tools and the relationship they develop with them over time. As they consistently use their tools, farmers develop increasing familiarity with their tools, which shapes their confidence and preference during use”. Line 516-519.

40. *538 You place only weather impacts on the tool in the environment category; is it possible to place other site factors there that are presently in the ‘task’ category, e.g. terrain conditions like slope, rocky soils that may wear a tool more quickly; arguably the latter is as impactful or more so on the wear of a tool than a rainy climate and rotting handle?*

**Response:** Thank you for this insightful observation. Terrain conditions were indeed identified by participants; however, they were primarily described in relation to task demands, such as physical effort and ease of tool use, rather than their impact on tool wear. For this reason, they were categorized under the “task” domain. The classification in this study reflects how participants framed their experiences. However, we acknowledge that terrain conditions may also influence tool degradation, and we have expanded the discussion to recognize this aspect and provide a more comprehensive perspective.

We added: “…Tool degradation may also result from mechanical wear caused by repeated contact with soil and its conditions including the presence of rocks, in addition to corrosion processes such as rust formation. In agricultural contexts, these abrasive interactions can contribute to the wear of tool edges, which may explain the frequent need for sharpening reported by participants”. Lines 592-596.

41. *559 “total tool length with the blade placed on the ground should be approximately….” Is the way to write this. Total is repeated and it is unclear.*

**Response:** We corrected the phrase as follows: “indicates that total tool length with the blade placed on the ground should be approximately at the user’s elbow height”. Lines 565, 566.

42. *579 conditions*

**Response:** corrected. Line 590.

43. *548 I feel that there may be an unstated conclusion here that you may want to bring out more explicitly, that a) it should be considered that tools are too heavy in design work going forward because they create too much fatigue but b) there may be reasons for a heavy tool in heavy soils, drawn from your participatory design effort. I wonder if a concluding sentence to the paragraph could say something like this.*

**Response:** Thank you for this valuable suggestion. We have added a concluding sentence to make the design implications more explicit, highlighting the need to balance tool weight to reduce fatigue while maintaining effectiveness under demanding soil conditions. Lines 553-555.

44. *562 Have you considered the literature regarding how Andean highland conditions may have produced human evolution towards shortened extremities (for heat conservation, or also unfortunately as a response to stress in childhood e.g. https://journals.plos.org/plosone/article?id=10.1371/journal.pone.0051795 )? It may be worth looking at, I don’t know if there has been a generational fading of this body shape pattern but it was definitely the case in my experience that Andean craft knitters had to deliberately lengthen the sleeves of sweaters for Northern tourists in past decades, if they used local arm models, sleeves were too short. This would make elbow height an unreliable guide and might produce tools that are too long and potentially more tiring; i.e. hard to swing; there may be an adaptedness of a shorter tool for shorter arms, in spite of bending tradeoffs. More generally considering whether the principles of tool measures from outside the region may be valid but the exact values might not be?*

**Response:** Thank you for the insightful comment. It prompted us to reflect more critically on the applicability of standard ergonomic recommendations in the Andean context. While our study did not directly assess variation in limb proportions, we have now incorporated relevant literature indicating that highland populations may present relatively shorter limb segments under conditions of environmental stress. This consideration is important, as it suggests that commonly used ergonomic references, such as tool length based on elbow height, may not fully capture local anthropometric variability. We have therefore revised the discussion to acknowledge that such standardized measurements, often derived from non-Andean populations, should be applied with caution and complemented by context-specific data.

It states as follows: “…Tool length is also relevant, the Canadian Centre for Occupational Health and Safety (CCOHS), indicates that total tool length with the blade placed on the ground should be approximately at the user’s elbow height [29]. While national anthropometric references values for Peru are limited, Escobar [30] reports estimated standing elbows height of approximately 103.1cm for men and 95.0 cm for women. However, anthropometric variability in Andean populations should also be considered. Evidence from highland Peru shows relatively shorter limb segments under conditions of environmental stress, reflecting growth trade-offs that prioritize essential functions over limb length [31]. This suggests that ergonomic guidelines derived from non-Andean populations may not be directly applicable and could result in tools that are suboptimal in length for local users”. Lines 564-573.

45. *579 again soil type might be an additional factor accelerating wear of the tool tip?*

**Response:** Thank you for the observation. We added this paragraph: Tool degradation may also result from mechanical wear caused by repeated contact with soil and its conditions including the presence of rocks, in addition to corrosion processes such as rust formation. In agricultural contexts, these abrasive interactions can contribute to the wear of tool edges, which may explain the frequent need for sharpening reported by participants. Line 592-596.

46. *605 I would say emerged not emerges (because in this sentence you are referring to the second question and therefore particular instance of results rather than generality)*

**Response:** Corrected. Line 620.

47. *613 Perhaps overstating the point, but I wonder if the conclusion here should be more nuanced in line with the other elements of the discussion such as in the next paragraph; they should perhaps ‘include technical criteria’ because the other principle you are demonstrating in the discussion is the local knowledge and adaptations of tool users? Rather than ‘adhering to’ which suggests that farmers may have a higher risk of getting it wrong. In my experience farmers who have enough experience to repair or modify tools often have a great deal of understanding of the particular problem they are trying to solve and are more often successful (and also line up well with technical criteria)*

**Response:** Thank you for this thoughtful observation. We have revised the wording to better reflect the role of farmers’ experiential knowledge, emphasizing that their practices can contribute and be supported by technical criteria, rather than suggesting strict adherence.

It states as follows: “…Another key factor is the possibility of performing self-maintenance, such as sharpening the tip, adjusting the handle, or replacing damaged parts, which reinforces a sense of control over the tool. These practices are grounded in farmer’s experiential knowledge and may be further supported by basic technical criteria, to enhance their effectiveness [34]”. Lines 625-629.

48. *622 this is a nice point and balances well against the rest of the paragraph. It might be a chance to stress the importance of participatory design (e.g. double diamond?) that ignores neither local knowledge nor the potential improvements from innovation with design criteria? – and perhaps a good transition to the next paragraph which deepens this argument?*

**Response:** Thank you for this valuable suggestion. We have expanded this paragraph to emphasize the importance of participatory design approaches. We added: “...These findings highlight the importance of participatory design approaches, such as the double diamond model, that integrate local knowledge with technical design criteria to ensure that innovations are both contextually appropriate and functionally effective”.

49. *633 would insert a comma “..older, locally crafted implements, …”*

**Response:** corrected. Line 652.

50. *655 I think you could insert a short phrase here defending the fact that you chose high frequency of use tools within a major and relatively universal Andean cropping activity (potato harvesting) --- eg. “Although we examined tools that are ubiquitous in the Andes and a corresponding activity that is one of the more common Andean smallholder farm activities, the small sample may limit…”*

**Response:** Thank you for this suggestion. We have revised the limitations section to better contextualize the study by first highlighting the relevance of the selected activity and tools within the Andean context, followed by a statement of the limitations regarding sample size

It states as follows**: “…**Although this study examined tools that are ubiquitous in the Andes within potato harvesting, one of the most common smallholder farming activities in this context, the sample was restricted to a single agricultural community…” Line 673-675.

51. *Figures – some errors in spelling:*

*Fig. 1: check these: terrain conditions, means of transport not mean, discomfort and bodily pain,*

**Response:** Corrected*.*

*Fig. 5: deficient tool not deficient; check that all bulleted entries on lists in boxes are capitalized on first word*

**Response:** Corrected
